# Dendritic Structures Functionalized with Boron Clusters, in Particular Carboranes, and Their Biological Properties †

**DOI:** 10.3390/pharmaceutics15082117

**Published:** 2023-08-10

**Authors:** Anne-Marie Caminade, Max Milewski, Evamarie Hey-Hawkins

**Affiliations:** 1 Laboratoire de Chimie de Coordination (LCC-CNRS) 205 Route de Narbonne, CEDEX 4, 31077 Toulouse, France; max.milewski@lcc-toulouse.fr; 2 LCC-CNRS, Université de Toulouse, CNRS, 31077 Toulouse, France; 3 Faculty of Chemistry and Mineralogy, Institute of Inorganic Chemistry, Leipzig University, Johannisallee 29, 04103 Leipzig, Germany; mm41cety@studserv.uni-leipzig.de

**Keywords:** boron clusters, carborane, dendrimer, boron neutron capture therapy (BNCT), cancer

## Abstract

The presence of a large number of boron atoms in boron clusters make them attractive tools for the treatment of cancer using boron neutron capture therapy (BNCT). Since the quantity of boron atoms present in the target cell directly affects the effectiveness of BNCT, the idea of gathering a high number of boron atoms in a single entity has emerged many years ago. In this perspective, using hyper-branched macromolecules such as dendrimers appears as an interesting solution. In this review, we will first present the synthesis of diverse dendritic entities (dendrimers, dendrons, and Janus dendrimers) that incorporate boron clusters, in particular carboranes, anywhere in their structure. Four parts of this review present the synthesis of dendrimers having boron clusters on the surface, or inside their structure, of dendrons and of Janus dendrimers, bearing boron clusters. Practically all these boronated dendritic structures were synthesized with the objective to study their biological properties, but in fact only a few of them have been tested against cancerous cells, and even a smaller number was tested in BNCT experiments. The biological experiments are discussed in the fifth part of this review. A good efficiency is generally observed with the boronated dendrimers, even in animal models, with an increase in their mean survival time (MST).

## 1. Introduction

**Scheme 1:**
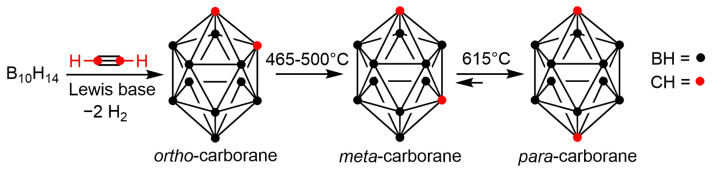
Synthesis of *ortho*-, *meta*-, and *para*-carborane (1,2-, 1,7-, and 1,12-*closo*-dicarbadodecaborane, respectively).

Boron clusters display a wide range of structures and intriguing properties. They have been used as materials, but also as antiseptic, antiviral, antitumor, and antifungal agents [1]. Carboranes (*closo*-dicarbadodecaboranes, also named carbaboranes) are a special class of boron clusters composed of two CR groups (R = mainly H) included in a B_10_H_10_ cluster [2]. The very first papers in the field of carboranes were published in 1963 [3,4]. They displayed the synthesis of *ortho*-carborane, by reaction of B_10_H_14_ with acetylene, in the presence of a Lewis base. Other isomers of B_10_C_2_H_12_ can be obtained by thermal rearrangement of the *ortho* isomer in an autoclave under inert conditions. The *meta* isomer is irreversibly obtained upon heating to 465–500 °C [5], whereas the *para* isomer is obtained at temperatures above 615 °C, but this latter reaction is reversible [6] (Scheme 1). 

**Scheme 2:**
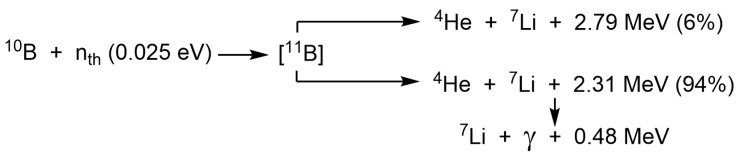
Reaction of ^10^B with low energy thermal neutrons used in BNCT.

Carboranes display a rich chemistry, at the level of the C−H groups, but also in some cases at the level of B−H groups. The chemistry of carboranes has been reviewed [7,8], in particular with emphasis on their biological properties [9]. Indeed, the large number of boron atoms present in these boron clusters made them desirable instruments for cancer treatment using boron neutron capture therapy (BNCT). The starting materials for BNCT are based on ^10^B, which is a nonradioactive constituent of the natural elemental boron. Irradiation of ^10^B with low energy thermal neutrons yields high linear energy transfer (LET) α particles (^4^He) and recoiling ^7^Li nuclei [10] (Scheme 2). 

In order to sustain a deadly ^10^B(n,α)^7^Li capture reaction, enough thermal neutrons must be captured by ^10^B (neutron capture cross-section is 3840 barn) to produce the high LET particles due to their constrained path lengths in tissues (5–9 μm). For this reason, a sufficient amount of ^10^B must be delivered to the tumor (≈20 μg/g or ≈10^9^ atoms/cell), making boron clusters appealing delivery systems for this purpose. In particular, sodium borocaptate (Na_2_B_12_H_11_SH) and *p*-boronophenylalanine (BPA), have been used in BNCT clinical studies [11]. 

Having more boron atoms in close proximity should increase the efficiency of BNCT. Dendrimers appear as a perfect solution. Indeed, dendrimers are hyper-branched macromolecules synthesized step-by-step, generally by a divergent process from a multifunctional core [12], having a large number of functions on their surface, which can be modified at will. The name “dendrimer” was coined by Donald A. Tomalia [13], to whom this review is dedicated, in gratitude for his inspiring work. He created the PAMAM dendrimers (PolyAMidoAMine), which are the most popular type of dendrimers [14]. 

Several reviews have already gathered some aspects of boron clusters linked to dendrimers, especially carboranes, but either the review is limited to the work produced by the authors of the review, and contains small molecules or cobalt derivatives (cobalt bis(dicarbollide) (or COSAN) derivatives) together with dendrimers [15,16,17], or dendrimers are only a part of the review [18,19,20], or only a small part of the review about dendrimers concerns boron clusters [21]. 

**Figure 1:**
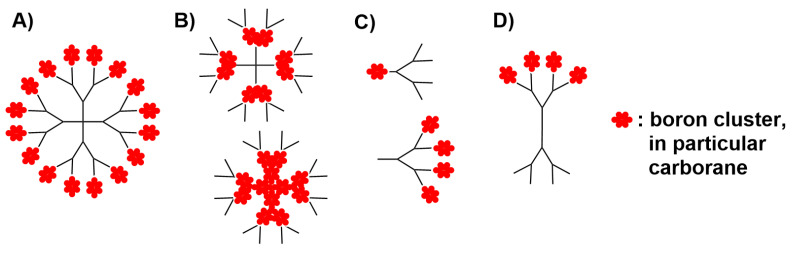
Different locations of boron clusters in dendritic compounds: surface of dendrimers (**A**), branches of dendrimers (**B**), core or surface of dendrons (**C**), and part of the surface of Janus dendrimers (**D**).

In this original review, we will present boron clusters, especially carboranes, anywhere in the structure of dendritic compounds, i.e., dendrimers and dendrons (dendritic wedges). We will consider only compounds in which the number of terminal functions is superior to the number of branches emanating from the core, as illustrated in Figure 1. This review is organized depending on the location of the boron clusters: on the surface of dendrimers (case A), at one or several layers inside the structure of dendrimers (case B), at the core or the surface of dendrons (case C), and on part of the surface of Janus dendrimers (case D). A last part will concern the biological properties (mainly but not exclusively for BNCT) of the dendritic compounds displayed in the previous parts.

## 2. Boron Clusters on the Surface of Dendrimers

Different types of dendrimers have been functionalized with boron clusters on their surface. This part will be organized depending on the type of the internal structure. The first, most widely used, and oldest examples concern PAMAM dendrimers. Other examples concern carbosilane dendrimers, dendrimers having a porphyrin or a phenylene core, and miscellaneous types of dendrimers.

### 2.1. Boronated PAMAM Dendrimers

**Scheme 3:**
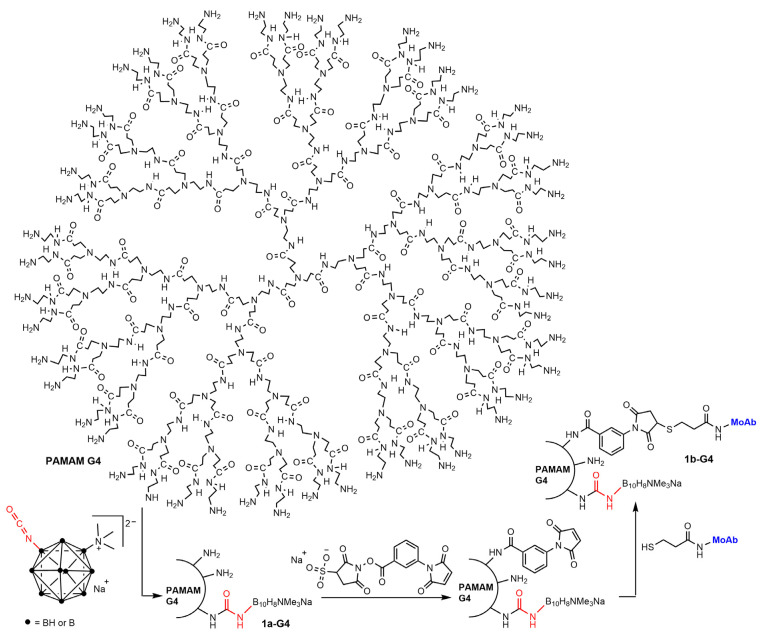
Full chemical structure of a generation 4 PAMAM dendrimer, and the stochastic grafting of B_10_ boron clusters and of a monoclonal antibody (MoAb) on its surface.

Second and fourth generation PAMAM dendrimers, bearing 12 (generation 2) or 48 (generation 4) NH_2_ surface functions have been functionalized with the isocyanato polyhedral borane shown in Scheme 3, affording the dendrimer **1a-G4** for the fourth generation. Analyses indicated that only some NH_2_ groups reacted, thus some of the remaining NH_2_ groups were used for the stochastic grafting of *m*-maleimidobenzoyl *N*-hydroxysulfosuccinimide ester, suitable for further reactions. For instance, the grafting to a monoclonal antibody (MoAb IB 16-6, which is directed against the murine B16 melanoma), afforded dendrimer **1b-G4**. A total of 8150 atoms of boron per molecule of antibody were measured, showing that in fact several dendrimers were grafted per MoAb unit [22]. 

Analogous types of reactions were carried out for the grafting of epidermal growth factor [23], folic acid and PEG (polyethylene glycol of variable length) [24], or vascular endothelial growth factor [25] to boronated PAMAM dendrimers. These dendrimers and related boronated PAMAM dendrimers bearing different types of MoAb were essentially synthesized for studying their biological properties, as will be discussed in Part 5 of this review. 

### 2.2. Boronated Carbosilane Dendrimers

**Scheme 4:**
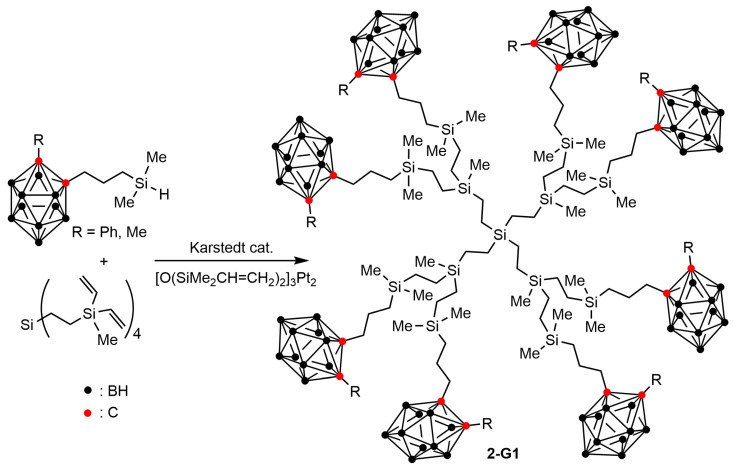
A first generation carbosilane dendrimer functionalized with eight carboranes.

Contrarily to PAMAM dendrimers, which have been functionalized stochastically with boron clusters, carbosilane dendrimers have been functionalized on all their terminal functions with only *ortho*-carboranes. Different types of reactions have been used. In a first example, the carborane was first functionalized with a methyl or phenyl group on one carbon atom, and then with an alkyl-SiH function, which adds to the vinyl groups of carbosilane dendrimers, using the Karstedt catalyst (platinum derivative of divinyl disiloxane). The first generation **2-G1** was isolated in about 60% yield (Scheme 4) [26]. 

**Scheme 5:**
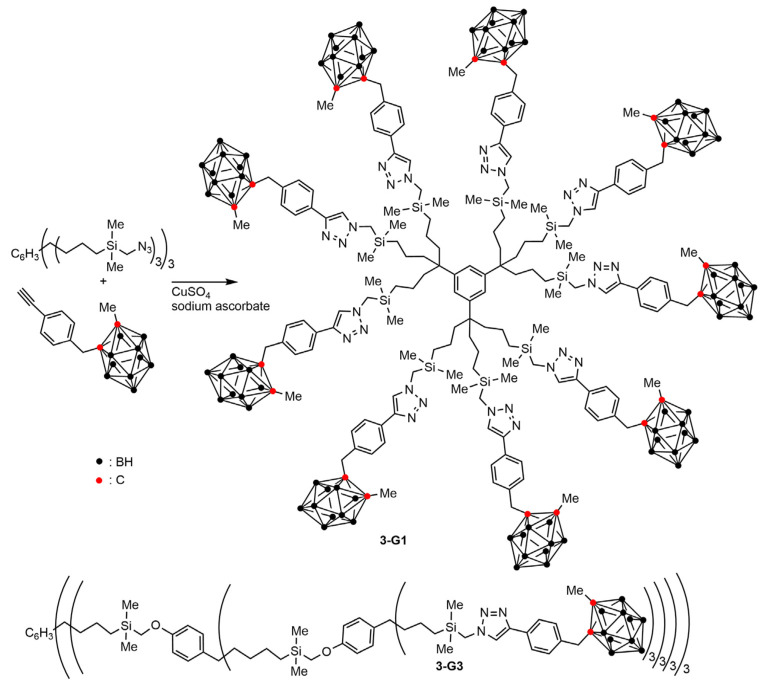
Carbosilane dendrimers functionalized with carboranes by CuAAC click reactions.

Larger boronated carbosilane dendrimers were synthesized by Copper(I)-catalyzed Huisgen-type azide-alkyne click cycloaddition reactions (CuAAC). The carborane was functionalized with a methyl group and an ethynyl group. The carbosilane dendrimers, from generation 0 to generation 3, were functionalized with azides as terminal functions. The CuAAC reaction afforded the expected dendrimers **3-G1** (nine carboranes) to **3-G3** (eighty-one carboranes) in about 80% yield. Scheme 5 displays the full structure of the first generation, and the linear structure with parentheses after each branching point for the third generation. Attempts to synthesize higher-generation dendrimers yielded insoluble materials. These dendrimers are particularly thermally stable, with only 10% mass loss at temperatures up to 392 °C for dendrimer **3-G3**. Low intensity emissions were observed between 280 and 480 nm with these carborane dendrimers [27].

**Scheme 6:**
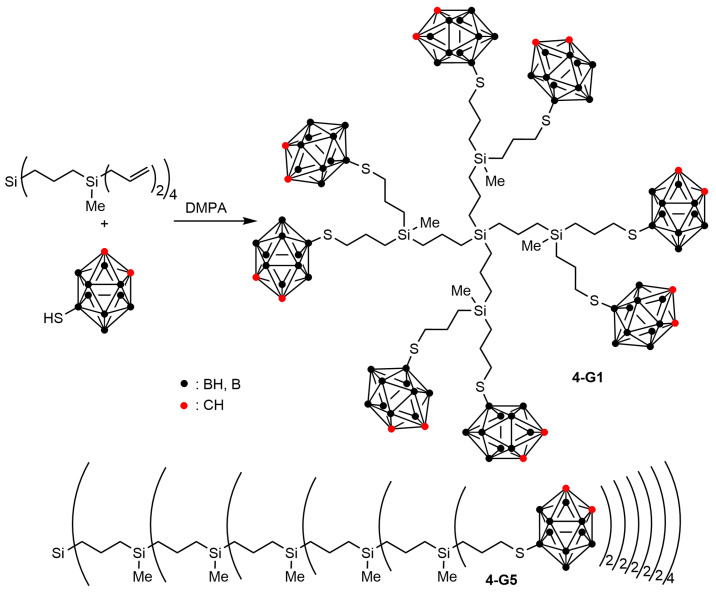
Carborane−carbosilane dendrimers functionalized by hydrothiolation.

A larger family of boronated carbosilane dendrimers was obtained from a carborane in which the carbon atoms were not used for the grafting to the dendrimers, contrarily to the previous cases. Hydrothiolation of carbosilane dendrimers capped with allyl groups and with 9-mercapto-*meta*-carborane initiated by UV irradiation in the presence of 2,2-dimethoxy-2-phenylacetophenone (DMPA) afforded carborane−carbosilane dendrimers of the zero, first, third, and fifth generations in about 60% yield. The full structure of the first generation **4-G1** and the linear structure with parentheses of the fifth generation **4-G5** are displayed in Scheme 6 [28]. The thermal properties and the rheological behavior of these dendrimers in relation with analogous polymers were recently compared [29].

### 2.3. Boronated Dendrimers Having a Porphyrin Core

**Scheme 7:**
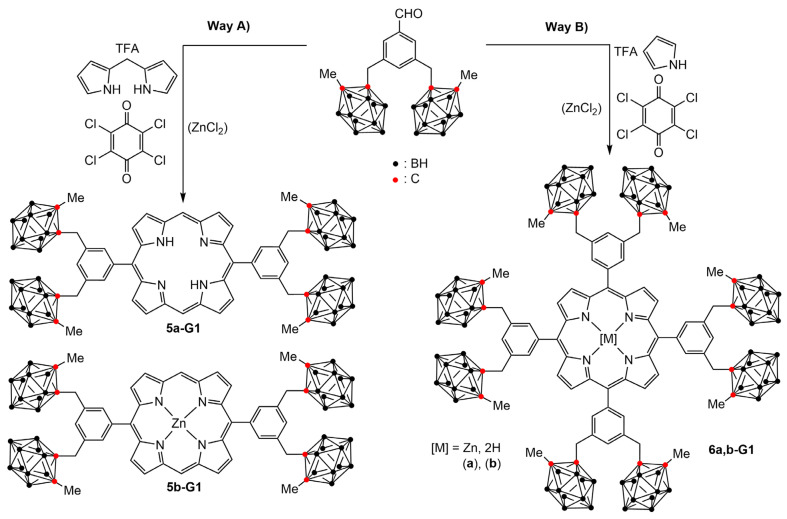
Porphyrin-cored first generation carborane dendrimers, either free (b) or complexing Zn (a).

A series of different carborane dendrimers bearing a porphyrin as core were synthesized as shown in Scheme 7. In these cases, a kind of divergent synthetic process was used as two carboranes were first linked to a benzaldehyde derivative. This was then used in condensation reactions with dipyrromethene in the presence of trifluoroacetic acid (TFA), to afford the porphyrin **5a-G1** functionalized with four carboranes, in 34% yield. Addition of ZnCl_2_ afforded the corresponding zinc complex **5b-G1** (Scheme 7, way A). Both the free porphyrin and the complex are insoluble in water, but they can be easily converted into the corresponding water-soluble *nido*-carboranylporphyrins by base degradation with pyridine/piperidine, followed by cation exchange with a Dowex resin [30]. An analogous process was applied to pyrrole instead of dipyrromethene, affording the porphyrin **6a-G1** functionalized with eight carboranes, isolated in 15% yield (Scheme 7, way B). The complexation of zinc (compound **6b-G1**) and the degradation to obtain water-soluble *nido*-carborane derivatives was carried out with compounds **6a,b-G1**, as was carried out for compound **5-G1** [31]. 

**Scheme 8:**
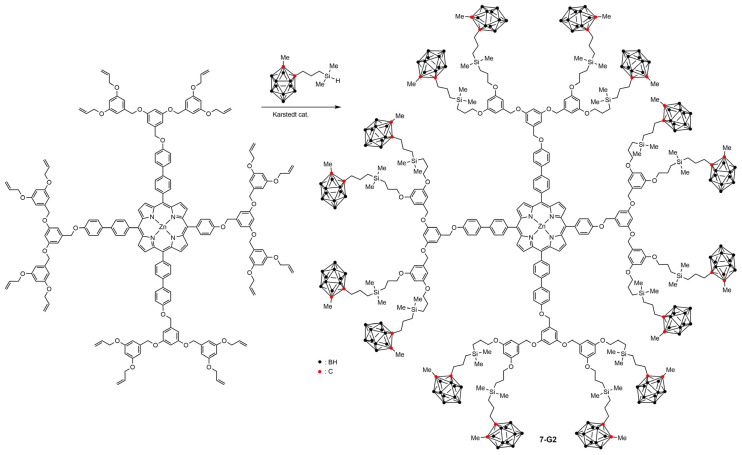
Synthesis of a generation 2 carborane dendrimer built from a porphyrin core. Generations 0, 1, and 3 were also synthesized.

Another series of porphyrin-cored carborane dendrimers was synthesized in a different way, by grafting the carboranes to the surface of porphyrin dendrimers. Generations 0 (four carboranes), 1 (eight carboranes), 2 (sixteen carboranes), and 3 (thirty-two carboranes) were synthesized by a regioselective hydrosilylation reaction on the allyl functions of the dendrimers, with carboranylsilanes in the presence of the Karstedt catalyst, as illustrated for generation 2 (**7-G2**) in Scheme 8. All the carboranyl−carbosilane dendrimers were isolated in good to excellent yields in the range of 60–88%. Studying the photoluminescence properties revealed that the photoluminescence characteristics of the porphyrin core are unaffected by the presence of carboranes [32]. 

### 2.4. Boronated Dendrimers Having a Phenylene Core

**Scheme 9:**
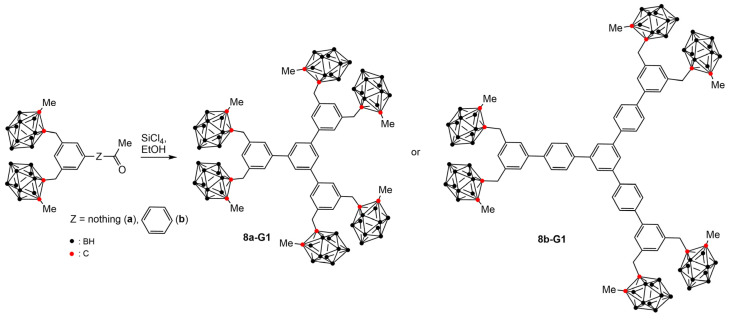
Trimerization of a ketone for the synthesis of first generation carborane dendrimers.

As in the case of the porphyrin core, boronated dendrimers having a phenylene core were synthesized by a convergent method or by a divergent process. In the first method, a trimerization reaction of arylketones bearing two carborane units mediated by silicon tetrachloride afforded the first generation dendrimers **8a,b-G1** in 55% (**a**) and 40% (**b**) yield, respectively (Scheme 9). In order to have water-soluble compounds, alcoholic NaOH was used to induce the removal of one of the vertex boron atoms to yield a monoanionic *nido*-carborane [33]. 

**Scheme 10:**
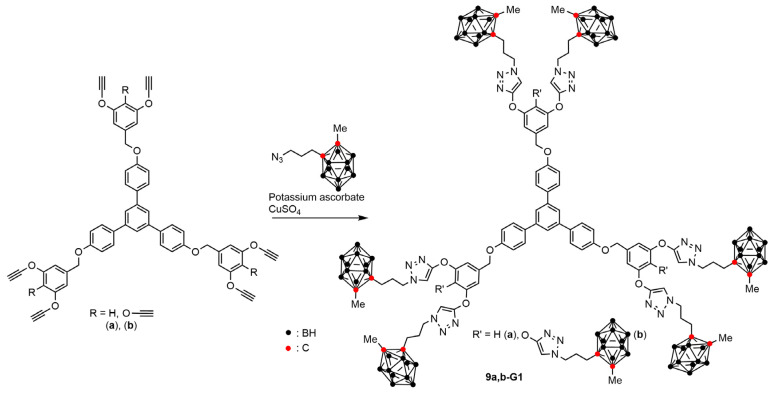
Synthesis of first generation carborane dendrimers obtained by CuAAC reactions, bearing either six (**9a-G1**) or nine (**9b-G1**) carboranes.

Another family of boronated dendrimers was obtained by CuAAC reactions between a dendrimer having six or nine alkyne functions and a carborane functionalized with an azide. Dendrimer **9a-G1** bearing six carborane units was isolated in 78% yield, whereas dendrimer **9b-G1** bearing nine carborane units was isolated in 76% yield (Scheme 10) [34].

**Scheme 11:**
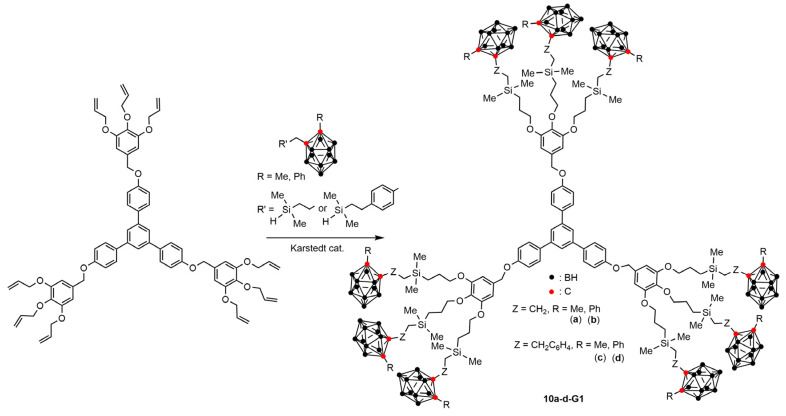
Hydrosilylation reactions for the synthesis of first generation carborane dendrimers **10a-G1** (Z = CH_2_, R = Me), **10b-G1** (Z = CH_2_, R = Ph), **10c-G1** (Z = CH_2_C_6_H_4_, R = Me), and **10d-G1** (Z = CH_2_ C_6_H_4_, R = Ph).

Four different carborane dendrimers (**10a-d-G1**) were obtained by hydrosilylation reactions between carboranes functionalized with a silane, and a dendrimer bearing nine allyl groups. They were isolated with yields in the range of 60–85% (Scheme 11). The removal of one boron vertex from each cluster leads to water-soluble macromolecules. Photoluminescent properties with high quantum yields, but depending on the nature of the clusters, were observed [35].

### 2.5. Miscellaneous Boronated Dendrimers

**Scheme 12:**
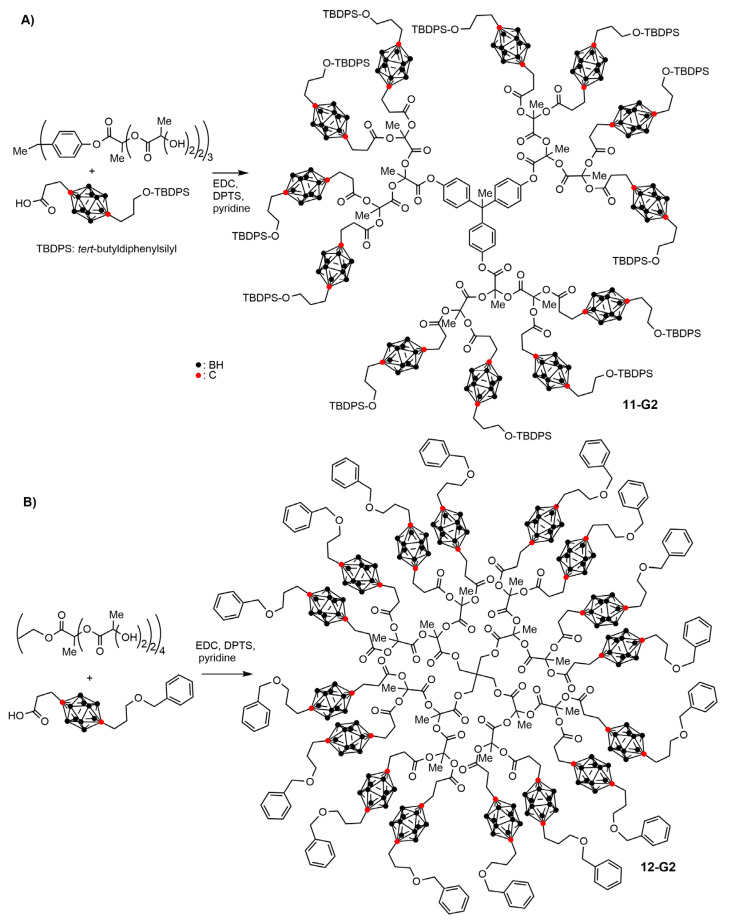
Synthesis of two types of *para*-carborane polyester dendrimers, functionalized on their surface with either TBDPS (**A**) or benzyl ether (**B**).

Different other types of carborane dendrimers were built from aliphatic polyester dendrimers, grown from 1,1,1-tris(hydroxyphenyl) ethane (Scheme 12A) or pentaerythritol (Scheme 12B) as core in the presence of EDC (1-[3-(dimethylamino)propyl]-3-ethylcarbodiimide) and DPTS (4-(dimethylamino)pyridinium-*p*-toluene sulfonate) as coupling agents. The dendrimers were isolated with yields ranging from 79 to 93%. Contrarily to all the previous cases which used *ortho*-carboranes, *para*-carboranes were used in these cases. The presence of TBDPS (*tert*-butyldiphenylsilyl) (**11-G2**) or benzyl ether (**12-G2**) as terminal functions enables the growth of new polyester branches as will be shown in Part 3.2. [36].

**Scheme 13:**
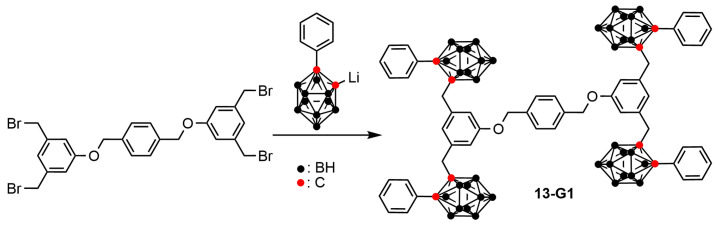
A small arylether dendrimer functionalized with carboranes.

A small carborane dendrimer (**13-G1**) was obtained from an arylether dendrimer and isolated in 82% yield (Scheme 13). As already indicated for other types of carborane dendrimers, water-soluble compounds were obtained by degrading the *closo*-cluster to the *nido*-cluster under basic conditions using KOH in EtOH. All these *closo-* and *nido*-carborane dendrimers show strong fluorescence emission in different solvents [37].

**Scheme 14:**
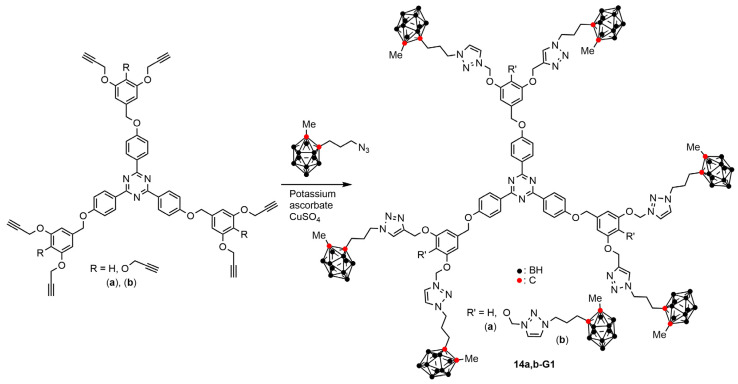
Triazine-cored arylether dendrimers functionalized with six (**14a-G1**) or nine (**14b-G1**) carborane units.

A series of triazine-cored small dendritic molecules containing three (not shown), six (**14a-G1**), or nine (**14b-G1**) peripheral carborane clusters were synthesized through CuAAC reactions and isolated in 75%, 71%, and 66% yield, respectively (Scheme 14). Thermal analyses demonstrated that dendrimers **14a-G1** and **14b-G1** maintained 53 and 54 wt% of residue at 700 °C, respectively [38].

## 3. Boron Clusters inside Dendrimers

As shown in the previous paragraphs, there are numerous examples of dendrimers functionalized on their surface by boron clusters, essentially *ortho*-carborane derivatives. Less research has been carried out on dendrimers functionalized in the internal structure by boron clusters. The very first example concerned the post-synthetic modification of the internal structure of hydrocarbon dendrimers, but in all the other cases, the borane clusters are introduced during the synthetic process.

### 3.1. Carboranes inside Hydrocarbon-Based Dendrimers 

**Scheme 15:**
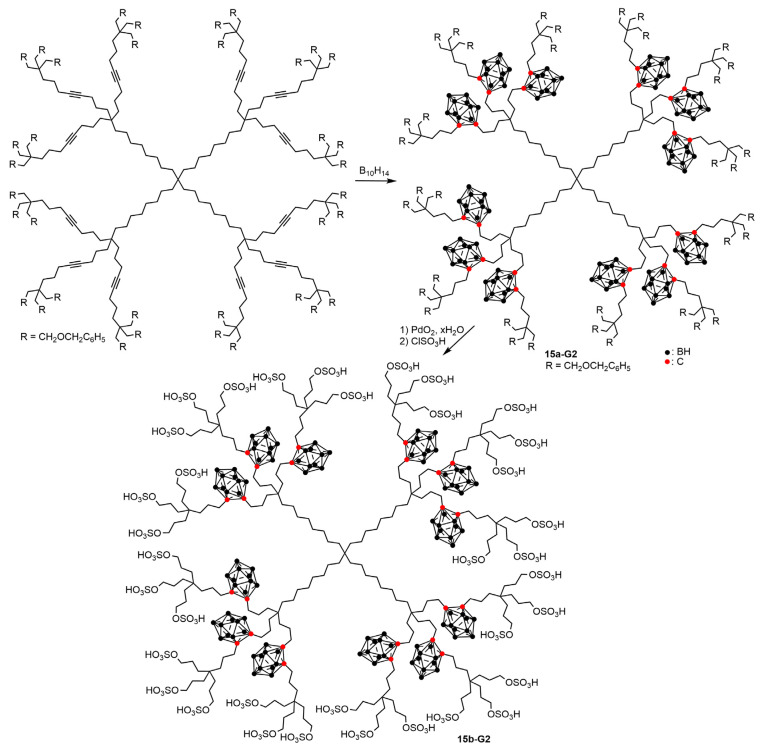
Addition of decaborane to internal alkynes of hydrocarbon dendrimers, and subsequent transformation of the terminal functions to obtain a water-soluble dendrimer.

Hydrocarbon dendrimers equipped with an internal layer of alkyne moieties were reacted with decaborane to afford different 1,2-dicarba-*closo*-dodecaboranes (Scheme 15), using the method shown in Scheme 1. This process was carried out with both, the first (four carboranes) and the second generation **15a-G2**, having twelve carboranes inside its structure, and being isolated in 77% yield. The terminal benzyl ether moieties were converted to peripheral alcohols, which were subsequently transformed to sulfate groups, to afford the water-soluble carborane dendrimer **15b-G2** [39]. The alcohol terminal groups were also converted to carboxylic acids [40].

The post-synthetic modification shown in Scheme 15 is an exception to obtain carboranes inside the structure of dendrimers. In all the other cases, dendritic branches are grown from functionalized carboranes on the surface of dendrimers.

### 3.2. Carboranes inside Polyester-Based Dendrimers

**Scheme 16:**
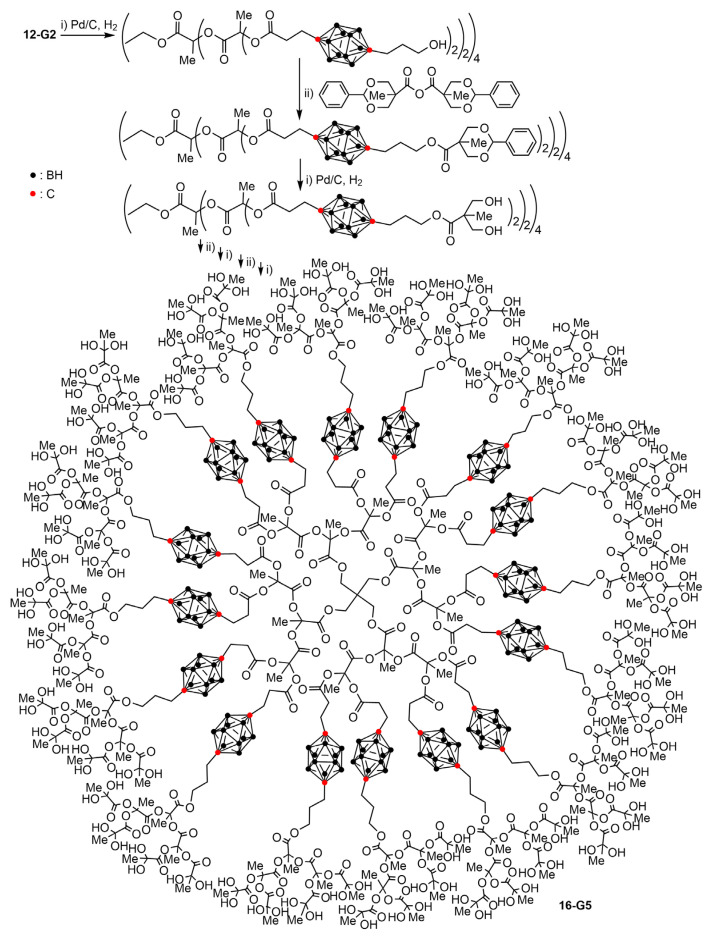
Step-by-step synthesis of dendrimer **16-G5** from dendrimer **12-G2** by repetition of step i) Pd/C, H_2_, and step ii) 2,2-bis(hydroxymethyl)propanoic acid.

Dendrimer **12-G2** (Scheme 12) possesses benzyl ether terminal functions, which were cleaved with Pd/C in the presence of H_2_, to obtain alcohol terminal functions, suitable for further divergent growth. Reaction with 2,2-bis(hydroxymethyl)propanoic acid (bis-MPA) was followed by deprotection with Pd/C in the presence of H_2_, to yield alcohol terminal functions. Repetition of both steps resulted in the growth of the dendritic branches, up to the fifth generation **16-G5** (Scheme 16) [36]. Analogous smaller dendrimers bearing four carboranes (**G2**) and eight carboranes (**G4**) were studied together with **16-G5** to determine their thermally-induced phase transition in aqueous media [41].

### 3.3. Carboranes inside Various Small Dendrimers

**Scheme 17:**
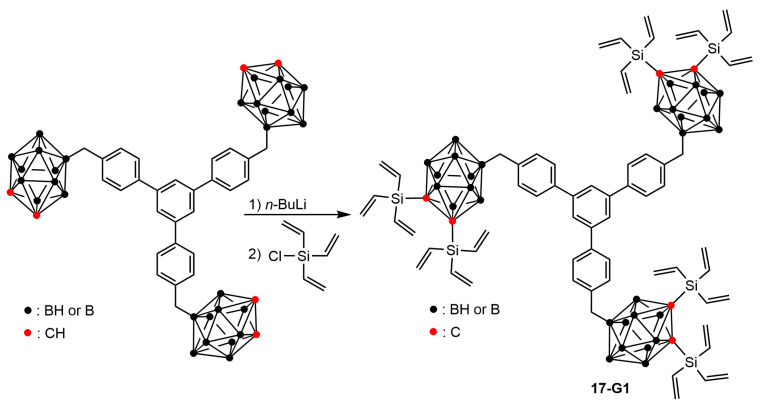
A small carbosilane dendrimer having carboranes inside its structure.

A small carborane dendrimer was first obtained by a cyclotrimerization reaction, analogous to the one shown in Scheme 9, and was then functionalized with trivinylsilane, to afford the phenylene-cored dendrimer **17-G1**, as shown in Scheme 17. This compound was isolated in 61% yield, and could be a precursor for the synthesis of larger carbosilane dendrimers [42].

**Scheme 18:**
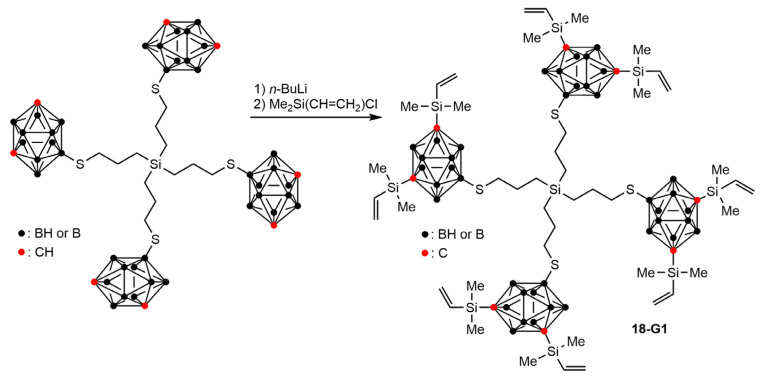
Another example of a small carbosilane–carborane dendrimer.

Another carbosilane dendrimer bearing four *meta*-carboranes linked through a B–S bond was functionalized with chloro(dimethyl) vinylsilane to afford the first generation dendrimer **18-G1**, isolated in 90% yield (Scheme 18) [43].

**Scheme 19:**
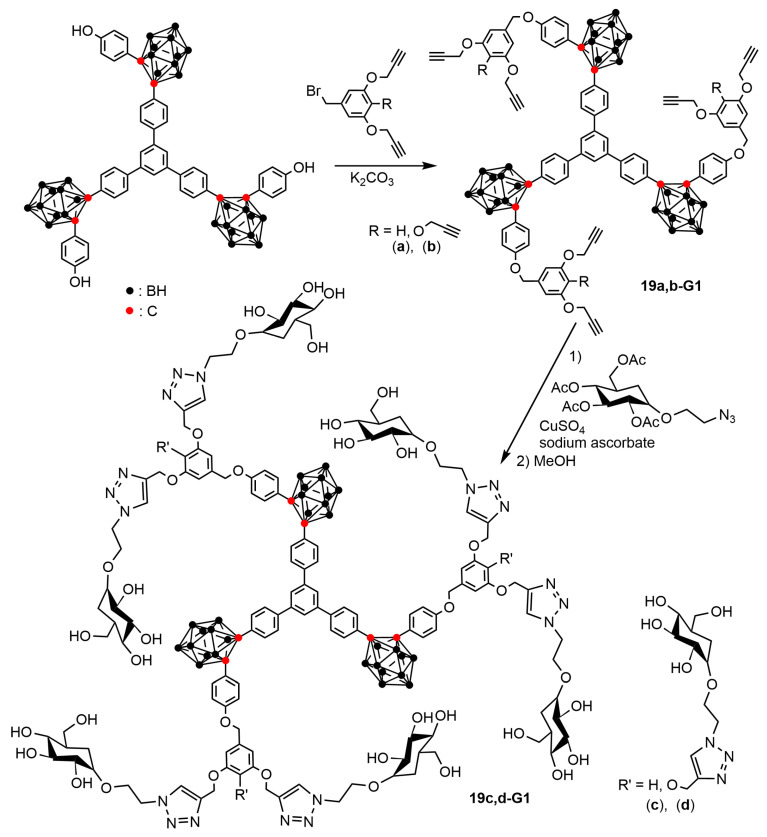
Dendrimer functionalized with carborane and either 6 (**19c-G1**) or 9 (**19d-G1**) glucose derivatives.

A recent paper displays the grafting of two or three alkynes per carborane on a phenylene-cored dendrimer to obtain dendrimers **19a-G1** and **19b-G1**, respectively. Dendrimers **19a,b-G1** were subsequently used in CuAAC reactions with a protected glucosyl azide, isolated in 55–60% yield. Deprotection in the last step afforded the glycodendrimers functionalized with three internal carboranes and six (**19c-G1**) or nine (**19d-G1**) glucose derivatives on the surface, isolated in 73–80% yield (Scheme 19) [44].

## 4. Dendrons and Dendritic Structures Functionalized with Boron Clusters

Even if the largest number of dendritic structures functionalized with boron clusters are dendrimers, the very first examples concerned dendrons having a single carborane at the level of the core. 

### 4.1. Boron Clusters at the Core of Dendrons

**Scheme 20:**
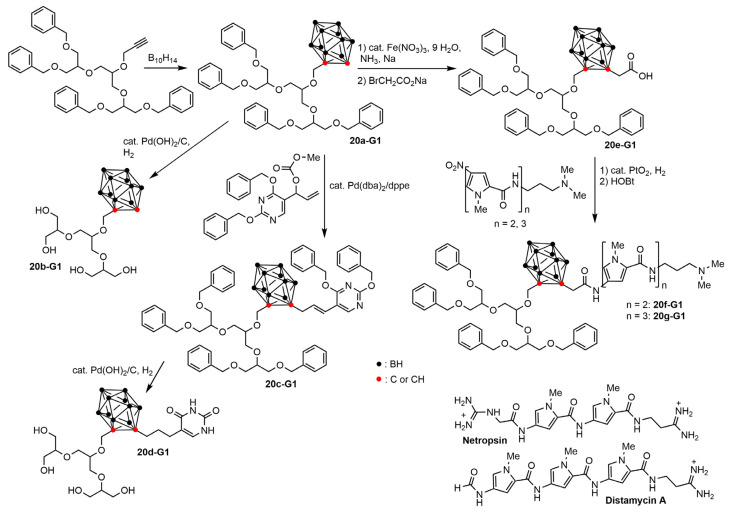
Examples of polyether dendrons with one carborane attached to the core and a variety of additional functionalities.

Small polyether dendrons (generations 0 and 1) bearing benzyl groups on the surface and an alkyne function at the core were reacted with decaborane, to afford dendron **20a-G1** for the first generation, isolated in 47% yield (Scheme 20). The benzyl groups were removed by hydrogenation in the presence of Pd(OH)_2_ on charcoal under acidic conditions in ethanol to afford the deprotected and water-soluble dendron **20b-G1** (solubility 5.44 mol/L), in 63% yield [45]. The presence of a C–H function on the carborane cluster permitted the performance of different types of functionalizations. In a first attempt, a protected uracyl moiety was grafted to **20a-G1** via a palladium-catalyzed reaction with Pd(dba)_2_/dppe (dba = dibenzylideneacetone, dppe = 1,2-bis(diphenylphosphino)ethane), to afford dendron **20c-G1** in 47% yield (Scheme 20). Hydrogenation of dendron **20c-G1** with palladium hydroxide on charcoal in ethanol afforded the fully deprotected and hydrogenated dendron **20d-G1** in 75% yield [46].

In a second attempt, netropsin and distamycin A analogues were grafted to the carboxylic acid dendron **20e-G1**, obtained by coupling the CH of the carborane of dendron **20a-G1** with BrCH_2_CO_2_Na. Netropsin and distamycin A (structure in Scheme 20) are natural products, possessing two or three methylpyrrole moieties, respectively. Both compounds are able to bind to the minor groove of double helical DNA. Their analogues were grafted first by reduction of the nitro group to the corresponding amine, followed by condensation with dendron **20e-G1**, using HOBt (hydroxybenzotriazole) and DCC (N,N′-dicyclohexylcarbodiimide) to give the desired products **20f-G1** and **20g-G1** both isolated in 65% yield (Scheme 20) [47]. 

**Scheme 21:**
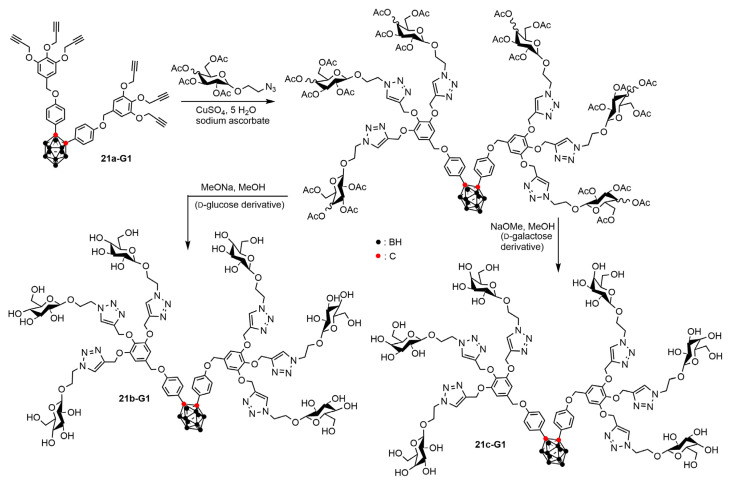
Carborane dendrons functionalized with D-glucose (**21b-G1**) or D-galactose (**21c-G1**) derivatives.

Another series of dendrons having a carborane at the core was synthesized by CuAAC reaction between carborane-cored dendrons bearing three or six alkyne groups (dendron **21a-G1**, with six alkyne groups, Scheme 21) and an azide linked to two types of carbohydrates, which are derivatives of protected D-glucose or protected D-galactose. These dendrons were isolated in moderate yields (52–54%). Deprotection of the alcohols with NaOMe in MeOH afforded dendrons functionalized with three (not shown) or six D-glucose (dendron **21b-G1**) or six D-galactose moieties (dendron **21c-G1**), in good yields (86–93%) (Scheme 21). Only dendrons functionalized with six carbohydrates (**21b-G1** and **21c-G1**) were fully soluble in water, contrarily with those functionalized with three carbohydrates [48]. 

**Scheme 22:**
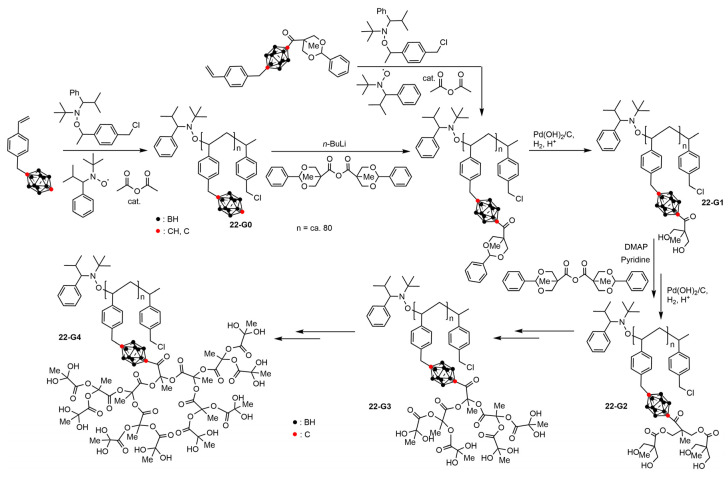
Synthesis of carborane polymers, followed by the growth of dendritic branches.

Special types of dendritic structures are the “dendronized polymers”, which are constituted by dendrons grafted to or synthesized on a linear polymer [49]. Two different approaches were used to obtain dendronized carboranyl polymers, using in both cases *para*-carboranes. In the first approach, the *para*-carborane monomer functionalized with a styrene group was polymerized by employing a nitroxide-mediated polymerization (NMP) strategy, using an alkoxyamine initiator, a catalytic amount of free nitroxyde radical and acetic acid in chlorobenzene to afford the polymer **22-G0**. In order to synthesize the dendrons stepwise, the polymer was first treated with n-BuLi and then with an excess of benzylidene-protected anhydride of bisMPA shown in Scheme 22. About 70% of the carboranes were functionalized in this way. Finally, removal of the benzylidene protecting groups was carried out using a Pd-catalyzed hydrogenolysis with PdOH_2_/C/H_2_, followed by an acid-catalyzed deprotection using H_2_SO_4_, to afford the first generation **22-G1**. In the second method to obtain polymer **22-G1**, the starting compound was a carboranyl monomer already functionalized on one carbon atom with a benzylidene-protecting group, and on the other carbon atom with a styrene group. Polymerization using the NMP strategy obtaining the same type of polymer and subsequent deprotection were carried out as indicated above. This approach yields the dendronized polymer **22-G1** that contains 100% functionalized carboranes, as opposed to the previous method yield of 70%. Polymer **22-G1** obtained by both methods was reacted successively in an iterative esterification with the benzylidene-protected anhydride of bisMPA, DMAP (4-dimethylaminopyridine) and pyridine. The second step was the deprotection of the anhydride, as illustrated in Scheme 22. In both cases, the synthesis was carried out up to the fourth generation of the dendrons, i.e., up to the dendronized polymer **22-G4** [50]. 

### 4.2. Boron Clusters on the Surface of Dendrons

**Figure 2:**
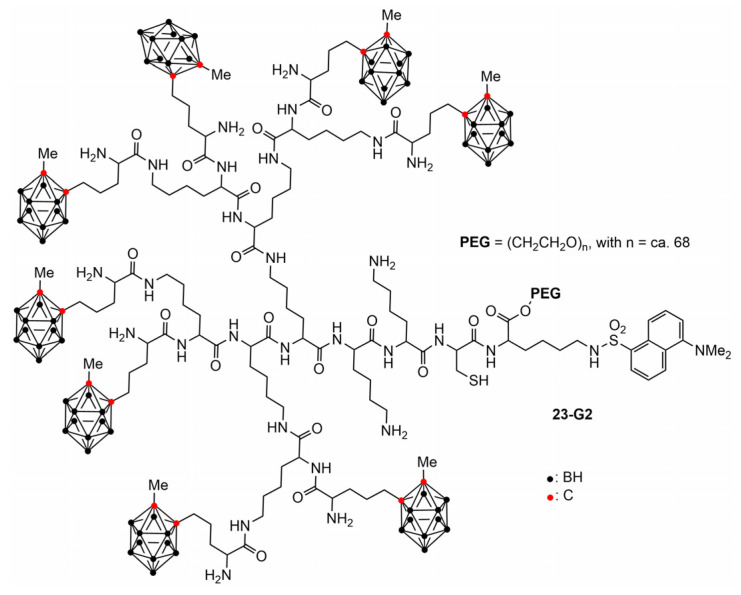
Structure of a poly-L-lysine carborane dendron.

A second generation poly-L-lysine dendron, obtained by solid state synthesis, was reacted in acylation reactions with (S)-5-(2-methyl)-l,2-dicarba-*closo*-dodecaborane(12)-1-yl)-2-amino pentanoic acid. The resulting dendron **23-G2** was isolated in 81% yield after cleavage and deprotection from the resin. It possesses a relatively long poly (ethyleneglycol) derivative composed of ca. 68 CH_2_CH_2_O groups at the core to increase the solubility of this dendron in water (Figure 2) [51].

**Scheme 23:**
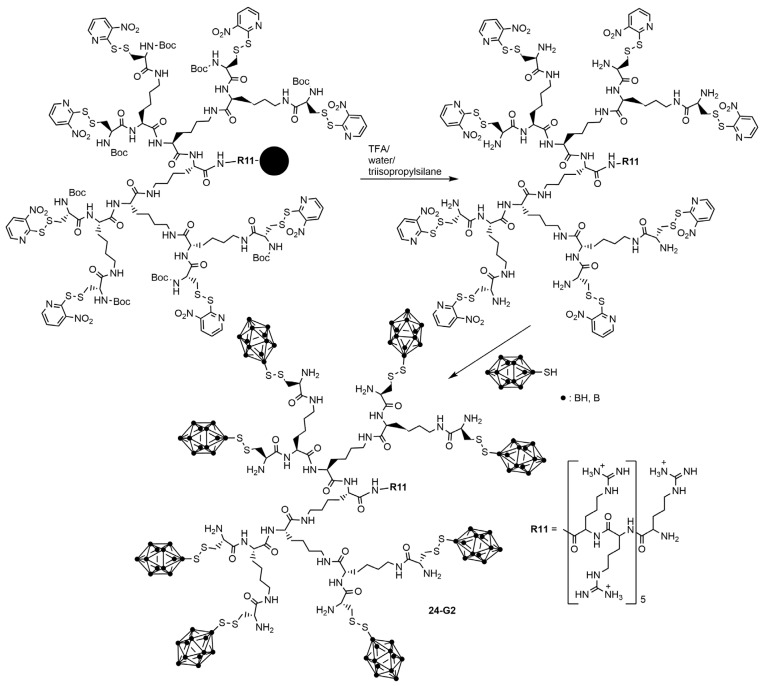
Another lysine-based peptide dendron functionalized with boron clusters on the surface.

Another type of peptide dendron functionalized with boron clusters on the surface was first obtained by growing different layers of peptides in the solid phase starting from R11, a cell-penetrating peptide composed of 11 arginine residues, linked to a resin. Prior to the reaction of the surface layer with a Boc-protected cysteine (Boc: *tert*-butyloxycarbonyl), three layers of lysine were constructed. Full deprotection of cysteine enabled also the cleavage of the dendron from the resin. Finally, eight mercaptoundecahydro-dodecaborate units were grafted on the surface, as illustrated in Scheme 23, to afford dendron **24-G2** isolated in 37% yield [52]. 

**Scheme 24:**
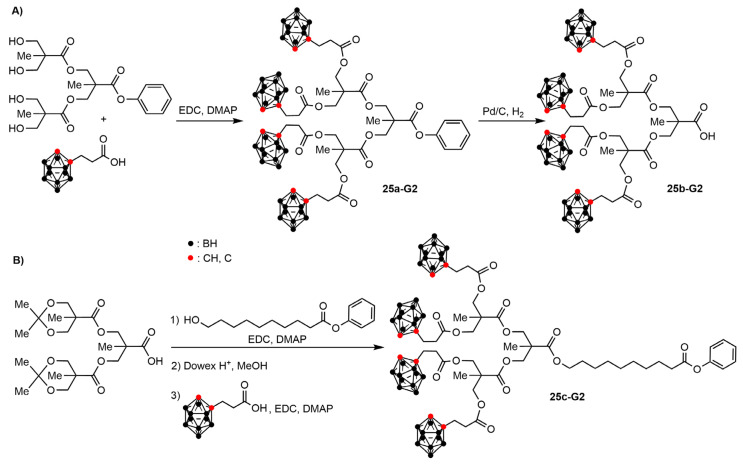
Several types of polyester carborane dendrons having either a carboxylic acid (**A**) or a benzyl-10-hydroxydecanoate (**B**).

Several types of small polyester dendrons bearing carboranes on their surface were synthesized by a fully divergent process or a mixture of divergent and convergent steps. A first example concerns a generation 2 polyester dendron reacted with a carborane functionalized with a carboxylic acid, in the presence of EDC and DMAP to afford dendron **25a-G2**, isolated in 75% yield (Scheme 24A). Deprotection of the benzyl group at the core was carried out with palladium on carbon, under H_2_, to afford dendron **25b-G2**. Attempts to react the carboxylic acid at the core with an alcohol failed, thus another way was used. The starting reagent was a surface-protected small dendron having a carboxylic acid as core, which was first reacted with benzyl-10-hydroxydecanoate, in the presence of EDC and DMAP. The next step was the deprotection of the surface with Dowex H^+^ in MeOH, followed by the grafting of a carborane functionalized with a carboxylic acid, as in Scheme 24A, to afford the dendron **25c-G2** (Scheme 24B) [53].

**Scheme 25:**
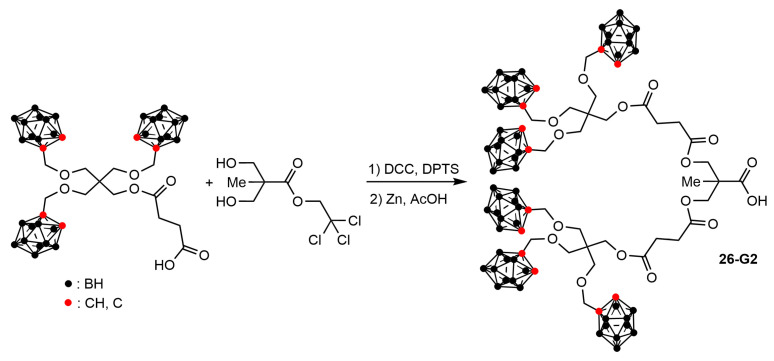
Polyester carborane dendron obtained by a convergent process.

Another type of polyester carborane dendron was obtained by a convergent process, which consisted first in adding the B_10_H_14_ cluster to a trialkinyl compound, for which the core was modified to graft a carboxylic acid. In the next step, the carboxylic acid was coupled to a diol, for which the core was modified in the last step to have again a carboxylic acid (see Scheme 25 for the last steps of the process). Dendron **26-G2** was obtained in this way in 90% yield. It is potentially suitable for further reactions, but the synthetic process was stopped at this step [54].

### 4.3. Carboranes on a Part of the Surface of Janus Dendrimers

**Scheme 26:**
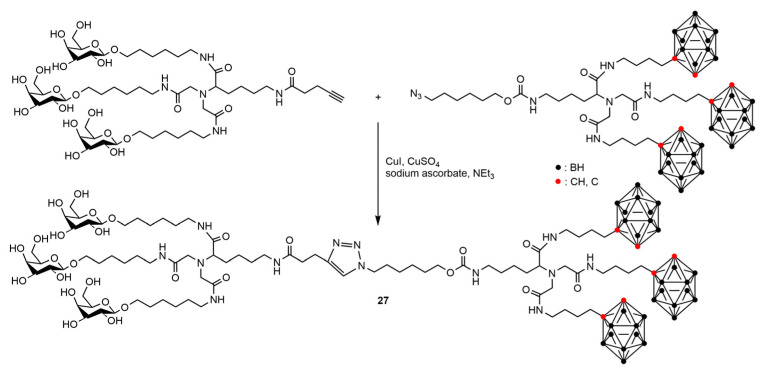
A small Janus dendrimer bearing carboranes on one side synthesized by CuAAC.

Janus dendrimers are constituted of two halves of different compositions at least on the surface [55]. They are frequently obtained by association of two dendrons through their core. Only very few examples of small Janus dendrimers functionalized with carboranes on a part of their surface have been reported. In a first example, a dendron bearing three galatosyl derivatives on the surface and an alkyne at the core was reacted with a dendron bearing three carboranes on the surface and an azide at the core. The association of both dendrons was carried out employing CuAAC, with CuI, CuSO_4_, sodium ascorbate, and NEt_3_ to afford compound **27,** isolated in 98% yield (Scheme 26) [56]. 

**Scheme 27:**
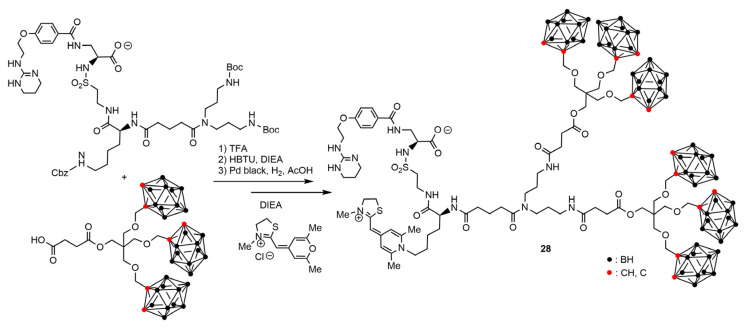
Last steps of the synthesis of a multifunctional Janus dendrimer.

The second example of a Janus dendrimer bearing carboranes on one side was obtained by a multi-step process, for which the last steps are shown in Scheme 27. The preliminary synthesis of a compound bearing an integrin ligand, two amines protected with Boc, and one amine protected with Cbz (carbobenzyloxy) was the starting point for the grafting of six carboranes. In the very last step, a monomethine cyanine dye for visible-light fluorescent imaging was grafted. Selective and sequential deprotection of the amines, using TFA, HBTU (2-(1H-benzotriazol-1-yl)-1,1,3,3-tetramethyluronium hexafluoro phosphate) as coupling agent, DIEA (N,N-diisopropylethylamine), and Pd black, H_2_ in acidic media, permitted the synthesis of the sophisticated Janus dendrimer **28** isolated in 25% yield [57].

## 5. Biological Properties of Dendritic Compounds Having Boron Clusters Somewhere in Their Structure

Practically all types of dendritic compounds incorporating boron clusters somewhere in their structure, as shown in this review, were synthesized to be used for BNCT. However, BNCT experiments were carried out only in a limited number of cases, due to the necessity to have a nuclear reactor laboratory in close proximity until only recently [58]. On the other hand, the anti-cancer properties of some of the dendritic structures shown in the previous paragraphs were tested in vitro or in vivo, without BNCT experiments. 

### 5.1. Anti-Cancer Properties of Dendritic Structures Functionalized with Boron Clusters 

Preliminary experiments concerned the possibility of localizing boron in the DNA of target (cancer) cells. For this purpose, analogs of netropsin and distamycin A that bind in the minor groove of double helical DNA were introduced into dendrons **20f-G1** and **20g-G1** (Scheme 20). It was shown that not only the presence of the methylpyrrole moieties, but also that of dendritic branches enhanced the efficiency of binding to the minor groove of DNA [47]. Other anti-cancer experiments were carried out mostly with dendrimers, and in particular with PAMAM dendrimers.

#### 5.1.1. Anti-Cancer Properties and In Vivo Localization of Boronated PAMAM Dendrimers 

**Figure 3:**
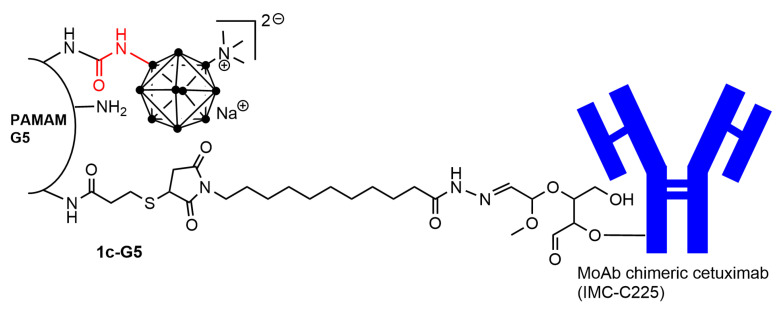
Schematic structure of boronated PAMAM **G5** functionalized with cetuximab.

The in vivo distribution of the PAMAM dendrimer **1b-G4** (Scheme 3) functionalized with a monoclonal antibody (MoAb) IB 16-6, which is directed against the murine B16 melanoma, was studied in normal and tumor-bearing mice carrying sub-cutaneous implants of the B16 melanoma. Unfortunately, a very limited quantity of **1b-G4** was found in the tumor [22]. Another type of MoAb, the chimeric cetuximab (IMC-C225) directed against the epidermal growth factor receptors EGFR and EGFRvIII, was grafted to the fifth generation of the boronated PAMAM dendrimer, as depicted in Figure 3, and using the method shown in Scheme 3. The in vivo localization of this dendrimer **1c-G5** after injection into the tumor of rats bearing intracerebral implants of F98 wild-type (F98_WT_) receptor (−) or EGFR gene-transfected F98_EGFR_ glioma cells demonstrated a much larger localization in F98_EGFR_ versus in F98_WT_ gliomas [59].

**Figure 4:**
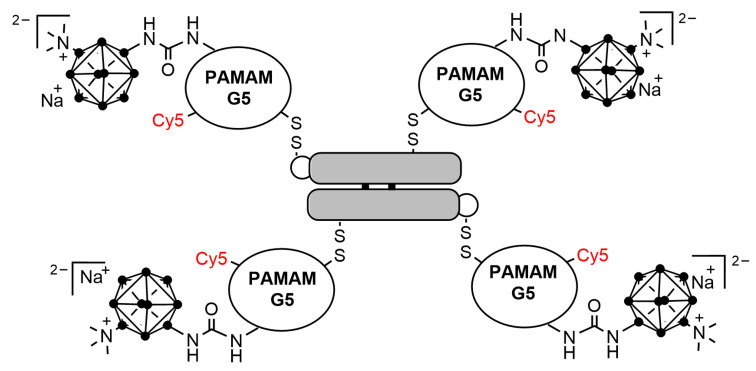
Schematic depiction of VEGF (gray rectangles) and its functionalization with several boronated fluorescent PAMAM dendrimers.

Another example of boronated PAMAM dendrimer was functionalized with the epidermal growth factor (EGF), since the EGFR gene is found on the cell surface of the majority of high-grade gliomas. It was shown by electron spectroscopic imaging that the dendrimer penetrated in human malignant glioma U-343MG cell line by endocytosis, and accumulated in lysosomes [23]. The third generation boronated PAMAM dendrimer was also reacted with PEGs of varying chain lengths, functionalized on the end with folic acid for targeting the folate receptor on cancer cells. First experiments on KB cells demonstrated a receptor-dependent uptake. However, in vivo experiments in C57BL/6 mice bearing the folate receptor (+) murine 24JK-FBP sarcomas resulted in a slightly selective tumor uptake (6.0% of the injected dose, ID/g tumor), but unwanted high hepatic (38.8% ID/g) and renal (62.8% ID/g) uptake were also observed [24]. A vascular endothelial growth factor (VEGF) was grafted to a boronated fifth generation PAMAM dendrimer, with the aim of targeting upregulated VEGF receptors (VEGFR), which are overexpressed on tumor neovasculature. This dendrimer was also tagged with a near-IR Cy5 dye for bioimaging. Several dendrimers were grafted per VEGF, as illustrated in Figure 4. Imaging of 4T1 mouse breast carcinoma revealed selective accumulation of VEGF-boronated dendrimer/Cy5, particularly at the tumor periphery where angiogenesis is most active [25]. 

#### 5.1.2. Anti-Cancer Properties and In Vivo Localization of Other Boronated Dendrimers

The biodistribution of the *nido*-carboranylporphyrins issued from **5a-G1** (free) and **5b-G1** (complexing Zn, see Scheme 7) were studied in BALB/c mice bearing EMT-6 mammary tumors. Both compounds were found to be effective tumor localizers, and the zinc(II) *nido*-complex was found to deliver 1.2–1.7 times higher amounts of boron to tumors than the free *nido*-compound [30]. The other *nido*-carboranylporphyrins issued from **6a-G1** and **6b-G1** were readily taken up by human glioblastoma T98G cells in culture, and were localized preferentially in the cell lysosomes. The metal-free *nido*-compound was found to accumulate to a higher extent in these cells compared with its zinc(II) *nido*-complex analog [31]. 

The dendrimer built from a phenylene core and functionalized with nine carboranes (**9b-G1**, Scheme 10) was tested toward human liver cancer cells (SK-Hep1). This dendrimer accumulated in the SK-Hep1 cancer cells in a concentration dependent manner, the highest accumulation being observed at a 50 μM concentration in **9b-G1** [34]. The biological properties of the triazine-cored dendrimers shown in Scheme 14, having six (**14a-G1**) or nine (**14b-G1**) carboranes on the surface have been evaluated using MCF-7 breast cancer cells. It was shown that the toxicity increased as the number of peripheral carboranes increased. The half maximal inhibitory concentration (IC_50_) value was found to be 80.67 ng/mL for the largest dendrimer **14b-G1** [38]. Very recently, dendrimers having three carboranes inside the structure and six (**19c-G1**) or nine (**19d-G1**) glucose moieties as surface functions (Scheme 19) were evaluated against MCF-7 breast cancer cell lines. The glycodendrimer **19d-G1** having the highest number of glucose moieties has shown the highest cell death potential via apoptosis, with an IC_50_ value of 41.35 ng/mL (0.01 μM) that is 600 times higher than the cytotoxicity of the chemotherapeutic drug cisplatin (IC_50_ 6 μM) [44]. 

Besides dendrimers, dendrons bearing a single carborane at the core and functionalized with three or six D-glucose (**21b-G1**) or D-galactose (**21c-G1**) (Scheme 21) were tested against two cancer cell lines (MCF-7 human breast carcinoma cells and A431 skin cancer cells) and one normal cell line (HaCaT skin epidermal cell line). All dendrons showed higher cytotoxicities toward cancer cell lines than toward the normal cell line. Compounds bearing only three glucose or galactose moieties were found to be more cytotoxic than the dendrons **21b-G1** and **21c-G1** [48]. The trifunctional theranostic agent **28**, comprising six carboranes, a monomethine cyanine dye for fluorescent imaging, and an integrin ligand for efficient tumor targeting (Scheme 27), was first tested toward WM115 (human melanoma) and MCF-7 cell lines. Confocal microscopy images indicated that the Janus dendrimer **28** was localized mainly on the cell membrane and in the lysosomes of both cell lines. The same compound was then used in vivo in T-cell deficient nude mice, which were subcutaneously engrafted with WM115 cells. Images show that compound **28** was already localized in the tumor 30 min after injection. A biodistribution study displayed a consistently higher degree of accumulation of compound **28** in the tumor as compared to the other vital organs, namely, lungs, heart, liver, kidneys, and spleen [57].

### 5.2. BNCT Properties of Dendritic Structures Functionalized with Boron Clusters 

The large majority of dendritic structures used in BNCT experiments were based on PAMAM dendrimers, functionalized with boron clusters on the surface, generally together with tumor-targeting moieties. The early publications in the field were previously reviewed [18], whereas a more recent review concerned the current advancements in the field of BNCT, including one paragraph about dendrimers [60]. 

#### 5.2.1. BNCT Properties of Boronated PAMAM Dendrimers

A boronated PAMAM dendrimer, functionalized with an EGF was first tested toward F98 wild-type (F98_WT_) receptor (−) or EGFR gene-transfected F98_EGFR_ cells implanted into the brains of Fischer rats. Biodistribution studies after 24 h demonstrated that 33.2% of injected dose/g of boronated PAMAM−EGF was retained by F98_EGFR_ gliomas compared with 9.4% of injected dose in F98_WT_ gliomas. Boron concentrations in normal brain, blood, liver, kidneys, and spleen were at nondetectable levels (<0.5 ng/g). Rats were irradiated for BNCT 24 h after injection of boronated PAMAM−EGF in the tumor. Untreated control rats had a mean survival time (MST) of 27 ± 1 days, and irradiated controls (without dendrimer) had a MST of 31 ± 1 days. Rats treated with the boronated dendrimer−EGF and which received BNCT had a MST of 33 ± 2 days for animals bearing F98_WT_ tumors, and of 45 ± 5 days for animals bearing F98_EGFR_ tumors [61]. In another paper, two types of injections of the same dendrimer bearing EGF were tested, the normal intratumoral (i.t.) injection, and the convection enhanced delivery (CED). Boron concentrations in F98_EGFR_ gliomas were 22.3 and 11.7 μg/g following CED and i.t. injection, respectively. Based on these results, BNCT experiments were carried out on rats that received the dendrimer injected i.v., or not. The MST was 53 ± 13 days, compared to 31 ± 4 days for irradiated control animals [62].

The boronated dendrimer functionalized with cetuximab **1c-G5** (Figure 3) was also injected in rats bearing F98_EGFR_ or F98_WT_ gliomas, resulting in the mean boron concentrations of 92.3 ± 23.3 μg/g and 36.5 ± 18.8 μg/g, respectively. The MST of rats that received **1c-G5** and were irradiated for BNCT was 45 ± 3 days, compared to 25 ± 3 days for untreated control animals [63]. The same dendrimer was also given intracerebrally by CED or direct i.t. injection into the brains of Fischer rats stereotactically implanted with F98_EGFR_ cells. The corresponding MST after BNCT was 54.5 ± 4.3 days, whereas the MSTs of irradiated and untreated controls were 30.3 ± 1.6 and 26.3 ± 1.6 days, respectively [64]. Another paper comparing the efficiency of CED and i.t. injections demonstrated a doubling of the tumor boron concentration (22.3 μg/g for CED vs. 11.7 μg/g for i.t.) [65].

Another monoclonal antibody, L8A4, directed against the receptor EGFRvIII, seems to be a truly tumor-specific target. L8A4 was also linked to the boronated PAMAM dendrimer **1a-G4** or **1a-G5**. This compound was injected in rats bearing F98 wild-type receptor (−) or EGFRvIII human gene-transfected receptor (+) F98_npEGFRvIII_ glioma cells. After 24 h, the amounts of dendrimer retained by receptor (+) gliomas were 60.1% following CED and 43.7% following i.t. injection compared with 14.6% by receptor (−) tumors. BNCT was carried out 24 h after administration of the dendrimer. Rats that received the dendrimer had a mean survival time of 70.4 ± 11.1 days with 10% long-term survivors (>6 months) compared with 30.3 ± 1.6 and 26.3 ± 1.1 days for irradiated and untreated controls, respectively [66]. Another paper compared the efficiency of boronated PAMAM dendrimers linked to cetuximab (**1c-G5**) or L8A4 in a rat glioma composed of a mixture of cells expressing wild-type (F98_EGFR_) or mutant receptors (F98_npEGFRvIII_). After BNCT, the mean survival time was 36 ± 1 days for boronated PAMAM−L8A4 and 38 ± 2 days for boronated PAMAM−cetuximab alone, whereas it was 55 ± 4 days when using the mixture of both dendrimers [67]. A short review summarized the work carried out with both types of boronated dendrimer−MoAb [68]. 

#### 5.2.2. BNCT Properties of Diverse Boronated Dendritic Structures

Dendrimers of the **16-Gn** series (Scheme 16, n = 3, with four internal carboranes and thirty-two OH terminal functions, and n = 4, with eight or sixteen internal carboranes and sixty-four OH terminal functions) in THF solution were placed in a thermal neutron beam of a nuclear reactor. The characteristic gamma radiation signature at an energy of 480 keV, corresponding to boron neutron capture events was observed in all cases, indicating the possible use of these compounds for BNCT [36].

The small Janus dendrimer **27** (Scheme 26) was used to treat HepG2 cells (human liver cancer cell line) by irradiation with thermal neutrons. In these conditions, the cell killing efficiency of dendrimer **27** was 10-fold greater than that of the monomeric BSH (sodium borocaptate, Na_2_B_12_H_11_SH) [56].

Dendron **24-G2** (Scheme 23) was first added to malignant glioma cells and found to localize at the cell nucleus. It was also administered to nude mice bearing a brain tumor after injection of U87 DEGFR cells. With neutron irradiation, the group that was treated with **24-G2** showed a significant cancer killing effect, even when compared to the group that was treated with a 100 times higher concentration of monomeric BSH, illustrating both the effect of the targeting group and of the concentration in boron due to the dendron [52]. 

## 6. Conclusions

The inclusion of boron clusters anywhere in the structure of dendrimers or dendrons has generated a rich chemistry, as illustrated in a large part of this review. About half of the dendritic structures synthesized were of first generations, but larger compounds were also synthesized, up to the fifth generation in some cases. Clearly, the small generations are easier to synthesize and to purify, but they accommodate a small number of boron clusters. On the contrary, high generations need time and money to be synthesized, their purity is sometimes questionable, but they can accommodate a large number of boron clusters. Up to now, most of the boronated dendritic structures tested in biological experiments are based on large PAMAM dendrimers of generations 4 or 5. Thus, having a large quantity of boron, and the possibility of accommodating targeting entities seems to be the most suitable solution for practical uses of boronated dendrimers. 

The biological properties of some of these dendritic structures, essentially dendrimers have been assessed against cancer cells, and in some cases in BNCT experiments, generally carried out with mice or rats. The relatively low number of boronated dendritic structures tested is certainly due to difficulty in having a nuclear reactor laboratory in close proximity, until recently. A good efficiency of the boronated dendritic structures in vivo in BNCT is generally observed, with a noticeable increase in the mean survival time of the animals. The presence of multiple functional groups on the surface of dendrimers enables the grafting of a targeting peptide, together with the boron clusters, for an improved localization in the cancer cells, and thus a better efficiency of BNCT. However, there is generally no cure of the cancer in these experiments, in particular concerning brain tumors in animals. This is probably the reason why no BNCT clinical trials have been carried out up to now with boronated dendrimers. However, as several accelerator-based BNCT facilities have been constructed in recent years with many still being under construction and planning [69], there is certainly a demand of improved and selective BNCT agents in future. 

Besides dendrimers, other types of carriers of boron clusters are known, such as polymers [70], nanoparticles [71], or liposomes [72]. Polymers can be of natural origin, such as dextrans or glucose polymers, but the structure of polymers is less well defined than that of dendrimers, and their functional groups are less easily accessible. Nanoparticles are frequently composed of toxic metals or of polymer balls, whereas liposomes are quite large compared to the size of dendrimers, but several types of liposomes for drug delivery have been approved by the U.S. Food and Drug Administration (FDA) and European Medicines Agency (EMA) [73]. It should be noted that different types of dendrimers have been and are used in different clinical trials [74], but not in the case of boronated dendrimers until now. In the view of state-of-the-art for the delivery of boron clusters, dendrimers appear to have a large potential, but that needs to be further developed.

## Data Availability

No data available.
